# Transition to tenecteplase is associated with shorter door-to-puncture times: a retrospective study from the Lone Star Stroke consortium TNK registry

**DOI:** 10.3389/fneur.2026.1804177

**Published:** 2026-05-04

**Authors:** Anqi Luo, Sujani Bandela, Gretchel Gealogo-Brown, Mark P. Goldberg, Andrew Slusher, Reza Behrouz, Alibay Jafarli, Siddarth Prasad, DaiWai Olson, Maria Denbow, Mehari Gebreyohanns, Asmiet Techan, Chethan P. Venkatasubba Rao, Jane A. Anderson, Barbara Kimmel, Anette Ovalle, Michele Patterson, Sean I. Savitz, Salvador Cruz-Flores, Steven Warach, Lee Birnbaum

**Affiliations:** 1 Department of Neurology, The University of Texas Health Science Center at San Antonio, San Antonio, TX, United States; 2 Department of Neurology, The University of Texas Southwestern Medical Center, Dallas, TX, United States; 3 Baylor College of Medicine, Houston, TX, United States; 4 Department of Neurology, Saint Luke's Baptist Hospital, San Antonio, TX, United States; 5 Department of Neurology, University of Texas Health Science Center at Houston, Houston, TX, United States; 6 Department of Neurology, Texas Tech University Health Sciences Center El Paso, El Paso, TX, United States; 7 Dell Medical School, The University of Texas at Austin and Ascension Texas, Austin, TX, United States

**Keywords:** door to puncture time, ischemic stroke, tenecteplase (TNK), thrombectomy, thrombolytic

## Abstract

**Background:**

Intravenous thrombolytic (IVT) and mechanical thrombectomy (MT) therapies are the current standard of care for large vessel occlusion (LVO) stroke. Multiple studies emphasized the impact of time metrics on patient outcomes, particularly door-to-needle (DTN) and door-to-puncture (DTP) times. Tenecteplase (TNK) offers potential advantages over alteplase (ALT), including a simplified one-time bolus administration, which may reduce DTP time. Results suggest TNK is non-inferior to ALT in terms of clinical outcomes, but few large cohort studies have compared DTP time for patients receiving TNK vs. ALT prior to thrombectomy. This real-world study aimed to compare DTP times and discharge outcomes in patients treated with TNK vs. ALT before thrombectomy.

**Methods:**

Retrospective data were collected from three comprehensive stroke centers (CSCs) in Texas from October 2019 to November 2024 and included subjects that received both IVT and MT. Data were analyzed for DTP times and other time metrics.

**Results:**

Among 50 ALT and 89 TNK patients in our study cohort, the TNK group had significantly shorter DTP times of 80 min (62–96) compared to ALT times of 101.5 min (80–121), *P* < 0.001. No significant differences were found for door-to-imaging and imaging-to-needle times; however, needle-to-puncture times were significantly shorter with TNK 39 min (29–51) compared to ALT 55 min (43–77), *P* < 0.001. Both groups had similar favorable outcomes at discharge.

**Conclusions:**

Our Lone Star Stroke (LSS) TNK registry represents the real-world experience of academic CSCs in Texas. We demonstrated that transitioning to TNK is associated with shorter DTP times compared to ALT. These results are primarily due to shorter needle-to-puncture times and may be attributable to TNK's simplified single-bolus administration. Both TNK and ALT groups demonstrated high rates of favorable outcomes at discharge, but given its faster DTP time, TNK is likely a preferable option for LVO stroke patients requiring both IVT and MT.

## Introduction

Stroke ranks as the fifth leading cause of death in United States and remains a significant contributor to adult long-term disability (1, 2). Acute ischemic stroke (AIS) accounts for the majority of strokes (3) and the restoration of cerebral blood flow reperfusion is crucial to minimize severe neurologic deficits and reduce mortality. Both intravenous thrombolytic (IVT) and mechanical thrombectomy (MT) therapies are standard of care for large vessel occlusion (LVO) within 4.5 h of stroke onset, with MT being available up to for 24 h (4). Numerous studies have highlighted the significance of time metrics in patient outcomes, particularly door-to-needle (DTN) and door-to-puncture (DTP) times (5). A shorter cerebral reperfusion time is associated with improved long-term functional outcomes and reduced mortality in LVO patients, who have the highest risks of morbidities and mortalities and comprise a significant percentage of all AIS patients nationally (6).

Alteplase (ALT) was the first Food and Drug Administration-approved drug for treating AIS after demonstrating efficacy in the 1995 National Institute of Neurological Disorders and Stroke study (7). Due to its short half-life of less than 6 min, ALT is administered as a bolus dose followed by a 1-h infusion period through a dedicated intravenous catheter (8). Alternatively, Tenecteplase (TNK) is a modified form of ALT that only requires bolus administration due to a longer half-life of 20–24 min (9, 10). This extended half-life allows for a more time- and cost-effective therapy, with potential superior efficacy in treating AIS. On average, TNK costs about half that of ALT and has been seen in randomized studies to be superior in LVO patients (11, 12). Thus, many comprehensive stroke center (CSC) hospitals over the past 5 years have transitioned from ALT to TNK (13, 14).

While studies suggest that TNK is non-inferior to ALT in terms of clinical AIS outcomes, limited large cohort studies have compared DTP times between patients receiving TNK or ALT before thrombectomy (15). The Lone Star Stroke (LSS) TNK registry includes multiple comprehensive stroke centers across Texas and is composed of a diverse ethnic and socioeconomic population. Three of these comprehensive centers transitioned from ALT to TNK between October 2019 and July 2022. Our study aimed to compare DTP times and outcomes at discharge for thrombectomy patients treated with TNK vs. ALT. We hypothesize that patients who receive TNK, rather than ALT, have shorter DTP times due to enhanced drug delivery with one-time bolus administration.

## Methods

We retrospectively collected patient data from October 2019 to November 2024 at three CSCs in Texas (hospitals A, B, and C). These hospitals are part of the LSS TNK registry and transitioned from ALT to TNK at different times during the study period: hospital A in July 2022, hospital B in April 2021, and hospital C in October 2020. The data collection was restricted to patients who presented to the emergency department (ED) and underwent both IVT and MT. Subject data entered into the registry aligned with the stroke variables collected by participating centers using the Get-With-the-Guidelines (GWTG) case report form. Hospital C investigators managed data transmission, curation, and harmonization across all participating sites using the Information Data Exchange and Acquisition System (IDEAS), a centralized, HIPAA-compliant data hub under the University's Clinical Research Informatics Division. Data analyses were performed using GraphPad (GraphPad Software, Inc., Boston, MA, USA) Prism for MacOS. Because the retrospective data does not include patient identifiers, the study received an exempt status from the institutional review boards of all participating sites. The dataset was dichotomized by IVT type and analyzed using descriptive statistics. A *P*-value less than 0.05 was considered statistically significant.

The primary measure was DTP times between the ALT and TNK groups. Subjects were excluded if DTP time exceeded 180-min, thrombolytic was administered at an outside hospital, and/or AIS was inpatient. Out of the 160 patients who met the inclusion criteria, 21 patients were excluded because their DTP exceeded the 180-min limit (Supplementary Table S1 exhibits characteristics of these patients). Time metrics including door-to-imaging, imaging-to-needle, and needle-to-puncture times were evaluated using the Chi Square test, unpaired *t*-test, Mann–Whitney test. Due to thrombolytic transition during the COVID-19 pandemic, subjects who received ALT in the 6 months prior to March 20, 2020 (the national COVID-19 lockdown date) were compared with those in the 6 months following that date. This analysis aimed to determine whether the pandemic significantly influenced DTP times, specifically in the ALT group.

## Results

**Figure 1:**
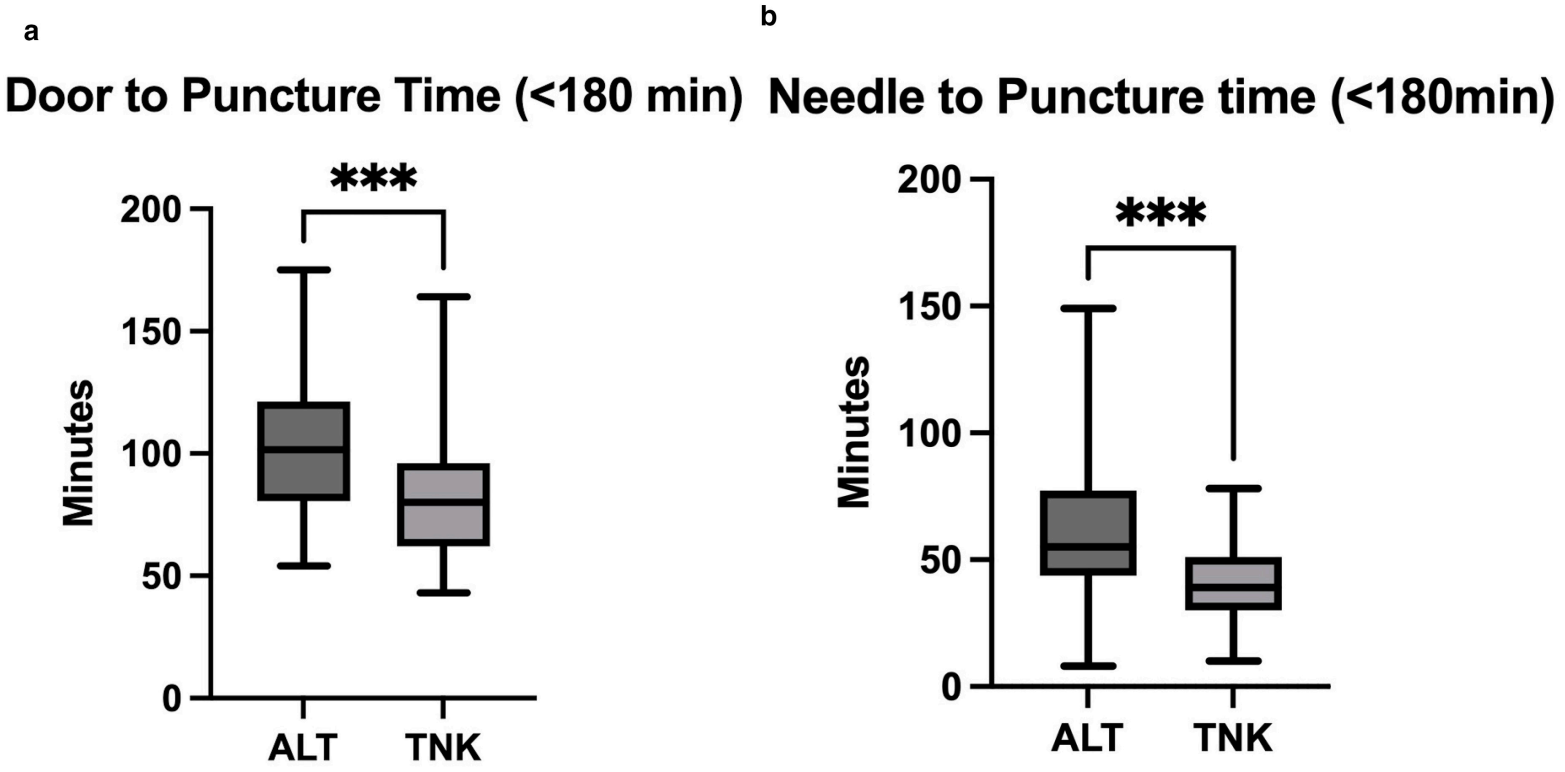
**(a)** Box plots and Median [IQR] comparing door-to-puncture time between ALT and TNK. ***Statistical significance. **(b)** Box plots and Median [IQR] comparing needle-to-puncture time between ALT and TNK. ***Statistical significance.

**Figure 2:**
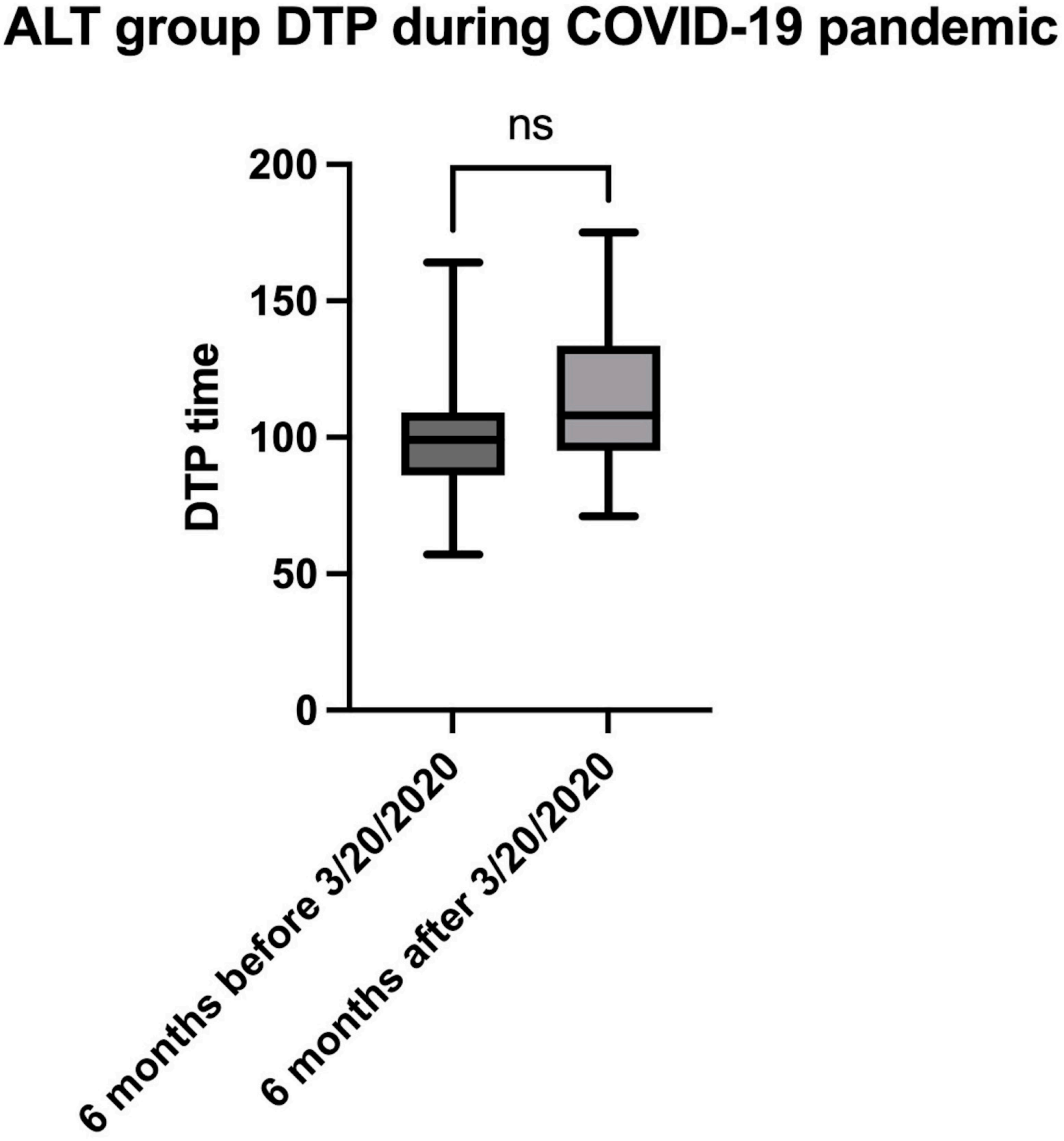
Box plots and Median [IQR] comparing subjects' door-to-puncture time between 6 months prior to March 20, 2020 (the national COVID-19 lockdown date), and the 6 months following that date.

The study cohort includes 50 patients who received IVT with ALT and 89 who received IVT with TNK. The mean ages, gender distributions, NIHSS scores, systolic blood pressure, BMI, Hispanic ethnicity, and initial blood glucose levels upon admission were comparable between the two groups (Table 1). Notably, both groups had a higher proportion of non-Hispanic individuals compared to Hispanic individuals. The TNK group displayed a similar median DTN time compared to the ALT group [36 min (28–47) vs. 39.5 min (33–48), *P* = 0.59]. However, DTP in the TNK group was significantly shorter compared to the ALT group [80 min (62–96) vs. 101.5 min (80–121), *P* < 0.001; Figure 1). The DTP times were further divided into multiple time metrics based on the stroke workflow, including door-to-imaging, imaging-to-needle, and needle-to-puncture. While no significant differences were observed in door-to-imaging and imaging-to-needle times, the needle-to-puncture time was significantly shorter with TNK compared to ALT, respectively [39 min (29–51) min vs. 55 (43–77) min, *P* < 0.001; Figure 1, Table 2]. We defined post-thrombolytic and thrombectomy intracranial hemorrhage as “any visible hemorrhage on post-procedure imaging, including subarachnoid hemorrhage, parenchymal hematoma, and/or intraventricular hemorrhage). There is no difference between TNK and ALT group (Table 1). Finally, both TNK and ALT groups demonstrated similar modified Rankin Scale (mRS) scores: 0–2 excellent outcomes and mRS: 0–3 favorable outcomes at discharge (Table 2, Supplementary Figures S2–S4). Over the year surrounding the national COVID-19 lockdown starting March 20, 2020, 15 patients received ALT in the preceding 6 months and 17 in the subsequent 6 months. No significant difference in DTP times was observed between these two periods of time [99 min (86–109) vs. 108 min (95–133); *P* = 0.33, Figure 2].

**Table 1:** Patient characteristics of the overall cohort.

Variable	TNK (*n* = 89)	ALT (*n* = 50)	*P*-value
Age, years (mean ± SD)	67.87 (15.21)	65.14 (15.03)	0.31
Gender, male % (*N*)	55.7 (49/88)	61.2 (30/49)	0.59
Hispanic %, (*N*)	30.3 (27/89)	38 (19/50)	0.45
Previous stroke history	12.3% (11/89)	10% (5/50)	0.79
Previous hypertension history	70.7% (63/89)	66% (33/50)	0.57
Previous diabetes history	24.7% (22/89)	30% (15/50)	0.55
Initial SBP, mmHg (mean ± SD)	154.7 (29.13)	154.6 (26.13)	0.98
Initial DBP, mmHg (mean ± SD)	88.10 (24.35)	91.12 (16.88)	0.44
Initial blood glucose, mg/dL (mean ± SD)	140.8 (63.84)	130.9 (36.72)	0.32
Initial NIHSS, median (IQR)	17 (12, 22)	16 (9, 21)	0.41
BMI	28.67 (6.33)	29.43 (7.24)	0.53
Any visible hemorrhage on post-procedure imaging	17.9% (16/89)	18% (9/50)	>0.99
Hospital A (patients)	13	16	
Hospital B (patients)	30	14	
Hospital C (patients)	46	20	

All analyses were performed using GraphPad Prism. T-test for parametric and Mann–Whitney test for non-parametric variables and Fisher's Exact Test for categorical variables.

^***^Statistical significance.

**Table 2:** Different time metrics and outcomes upon discharge.

Time metrics	TNK (*n* = 89)	ALT (*n* = 50)	*P*-value
Door-to-puncture time, median (IQR), min	80 (62, 96)	101.5 (80.5, 121.3)	< 0.001^***^
Door-to-needle time, median (IQR), min	36 (28, 47)	39.5 (33, 48)	0.59
Door-to-imaging time, median (IQR), min	9 (6.5, 14.5)	10 (5.7, 15.2)	0.36
Imaging-to-needle time, median (IQR), min	25 (18.5, 33.5)	30.5 (22.7, 38)	0.17
Needle-to-puncture time, median (IQR), min	39 (29, 51)	55 (43.7, 77.2)	< 0.001^***^
Excellent outcome mRS 0–2, %, (*N*)	26.5% (22/83)	31.8% (14/44)	0.54
Favorable outcome mRS 0–3, %, (*N*)	44.5% (37/83)	45.4% (20/44)	>0.99

All analyses were performed using GraphPad Prism. T-test for parametric and Mann–Whitney test for non-parametric variables and Fisher's Exact Test for categorical variables.

^***^Statistical significance.

## Discussion

Combined IVT and MT are recommended for eligible patients with LVO (4). The BRIDGE-TNK (Randomized Trial of Thrombectomy with vs. Without rhTNK-tPA in Stroke) study recently demonstrated that among patients with AIS due to LVO who presented within 4.5 h after onset, the percentage of patients with functional independence at 90 days was higher with TNK plus MT compared to MT alone (16). Furthermore, the Improving Reperfusion Strategies in Ischemic Stroke (IRIS) Collaborators analyzed individual participant data from six randomized trials comparing IVT (99% ALT) plus MT vs. MT alone in patients with LVO and concluded that the clinical benefit of IVT prior to MT is time-dependent, with earlier treatment associated with better outcomes (17). Additionally, the national GWTG database revealed that each 15-min increase in DTN time significantly affected patient outcomes, particularly for those receiving both IVT and MT, and every 30-min increase in DTP time was associated with a lower probability of discharge home (5). The abundance of time-dependent evidence for LVO triage within 4.5 h highlights the importance of efficient IVT without delay in DTP times (6, 18).

Our real-world study of three Texas CSCs that transitioned from ALT to TNK is associated with shorter DTP times, primarily because of a shorter needle-to-puncture time. This further supports the advantages of TNK transition. Consistent with our previously published overall IVT cohort, the TNK transition did not significantly change DTN time metrics in patients who presented with LVO. Both our TNK and ALT groups demonstrated faster DTN times compared to our previously published non-LVO patients and may be a result of the more severe and recognizable symptoms of LVO strokes, which prompts quicker decision-making for IVT (19). Regardless, it is important that TNK is not delayed for LVO stroke patients, which should translate into faster DTP times.

Our sample was obtained from CSC hospitals, which are likely to be better equipped to treat acute stroke (20). Because our study was limited to LSS CSCs that transitioned thrombolytic during the study period, we were better able to control for confounders as each hospital system may have different AIS triage protocols. For example, some CSCs utilized MRI, rather than CT, and others employed tele-stroke services, rather than in-house neurology. We reduced selection bias by excluding LSS CSCs that did not transition during the study period.

### Limitations

Limitations in our study include the transition from ALT to TNK during the COVID-19 pandemic which impacted stroke systems of care, especially during the lockdown. We showed a non-significant median DTP time for ALT, though 10 min longer, during the 6 months after the COVID-19 national lockdown. On the other hand, despite COVID-19, the CSCs in this study successfully transitioned to TNK which further highlights its safety and efficiency. Our previously published paper, which analyzed a larger patient population, also confirmed this assertion (21). Our primary functional outcome is discharge mRS rather than the standard 90-day mRS, which is a significant limitation. Discharge mRS is influenced by variable lengths of stay and does not capture delayed recovery or complications after discharge. Therefore, our conclusions focus primarily on workflow metrics (door-to-puncture times), and the functional outcome data should be interpreted as exploratory only. Previous study has demonstrated that IVT at a primary stroke center before transfer for MT can reduce infarction progression and potentially shorten the DTP time (22). Our study only included patients who presented to the emergency room and excluded transfer patients. Thus, our results do not extrapolate to the “drip/bolus and ship” models.

Also, our results are based on univariable/unadjusted analyses, and we acknowledge that potential confounders may have influenced the findings. Although treatment time metrics may improve over time, the three CSCs had been providing patient care based on the Target: Stroke model since 2011 (23), have established stroke workflows, and did not report any ongoing quality improvement projects during the study timeframe. All hospitals underwent comprehensive training before transitioning to TNK and maintained their existing workflows.

## Conclusion

Our LSS TNK registry represents the real-world experience of three academic CSCs in Texas. We demonstrated that transitioning from ALT to TNK is associated with shorter DTP times. These results are primarily due to shorter needle-to-puncture times and may be attributable to TNK's simplified single-bolus administration. Both TNK and ALT groups demonstrated high rates of favorable outcomes at discharge, but given its faster DTP time, TNK is likely a preferable option for LVO stroke patients requiring both IVT and MT. Future studies involving larger patient volumes are necessary to validate our findings, considering our moderate sample sizes, retrospective design and only descriptive statistics.

## Data Availability

The raw data supporting the conclusions of this article will be made available by the authors, without undue reservation.

## Supplementary material

Supplementary Table S1 Patient characteristics of the patients who were excluded. All analyses were performed using GraphPad Prism. T-test for parametric and Mann-Whitney test for non-parametric variables and Fisher's Exact Test for categorical variables. ^***^Statistical significance.

Supplementary Figure S1 Graphical Abstract.

Supplementary Figure S2 Compare the distribution of mRS at discharge between the ALT and TNK groups.

Supplementary Figure S3 mRS at discharge distribution bar (mRS 0–3 vs. 4–6).

Supplementary Figure S4 mRS at discharge distribution bar (mRS 0–2 vs. 3–6).
