# Supplementary figures and images for: Transition to tenecteplase is associated with shorter door-to-puncture times: a retrospective study from the Lone Star Stroke consortium TNK registry
Source: Front Neurol. 2026 May 4;17:1804177. doi: 10.3389/fneur.2026.1804177 (PMC13181930; doi:10.3389/fneur.2026.1804177)

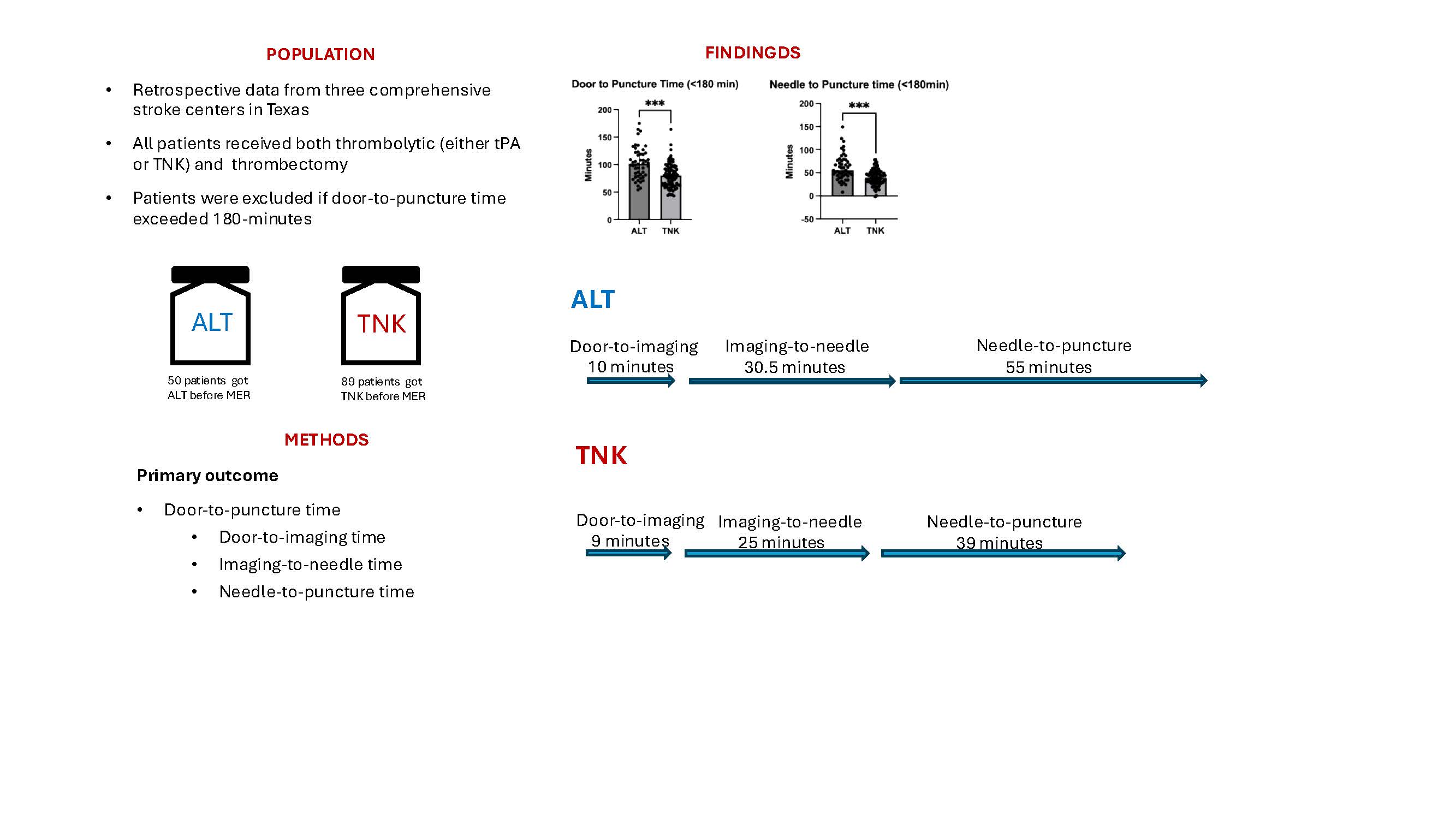

Supplement: Supplementary Table S1 — Patient characteristics of the patients who were excluded. All analyses were performed using GraphPad Prism. T-test for parametric and Mann-Whitney test for non-parametric variables and Fisher's Exact Test for categorical variables. ***Statistical significance. [file Image_1.jpeg]

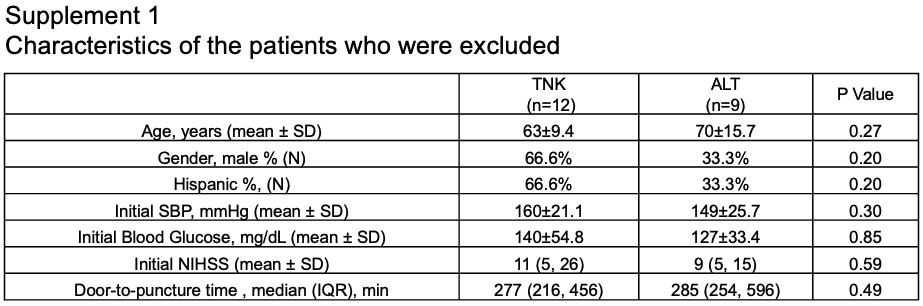

Supplement: Supplementary Figure S1 — Graphical Abstract. [file Image_2.png]

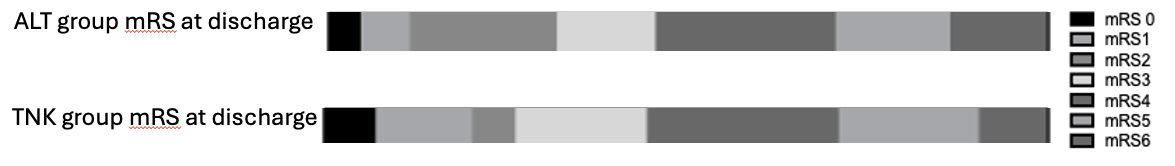

Supplement: Supplementary Figure S2 — Compare the distribution of mRS at discharge between the ALT and TNK groups. [file Image_3.png]

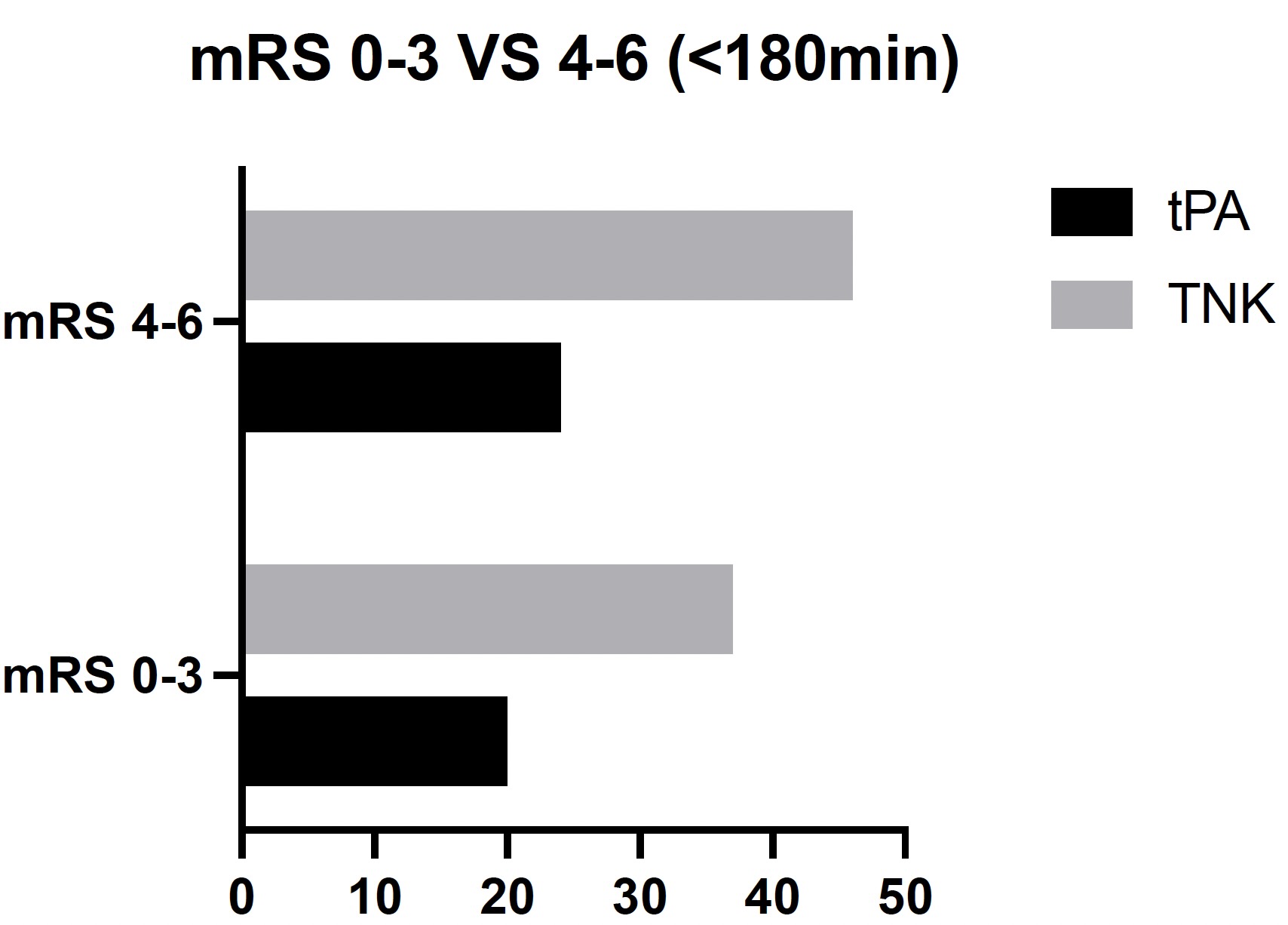

Supplement: Supplementary Figure S3 — mRS at discharge distribution bar (mRS 0–3 vs. 4–6). [file Image_4.jpeg]

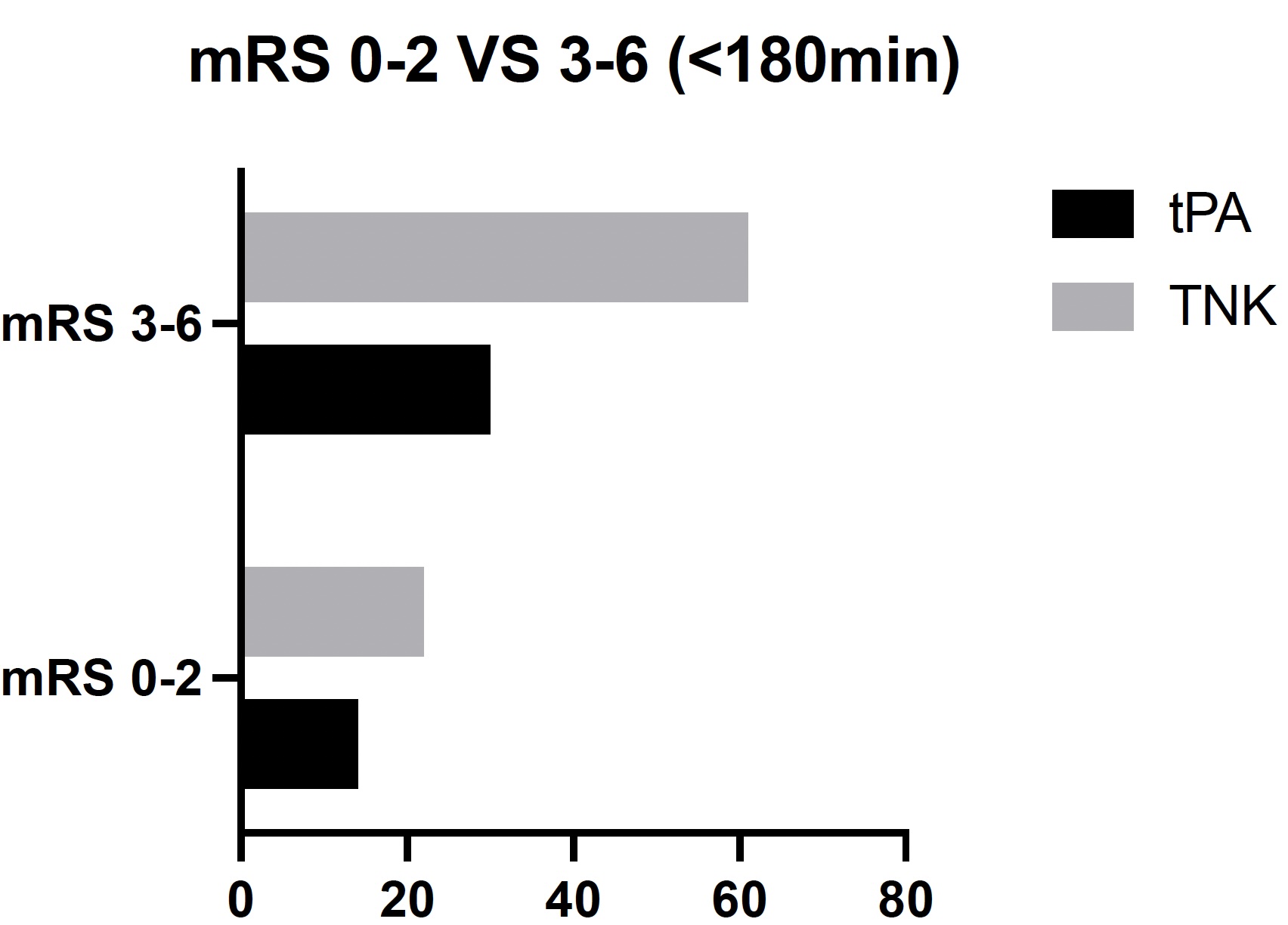

Supplement: Supplementary Figure S4 — mRS at discharge distribution bar (mRS 0–2 vs. 3–6). [file Image_5.jpeg]
